# Study of the influence of phantom material and size on the calibration of ionization chambers in terms of absorbed dose to water

**DOI:** 10.1120/jacmp.v7i3.2264

**Published:** 2006-08-24

**Authors:** Mehenna Arib, Toufik Medjadj, Youcef Boudouma

**Affiliations:** ^1^ Secondary Standard Dosimetry Laboratory Nuclear Research Centre of Algiers 2 Bd Frantz Fanon BP 399 Alger Gare Alger 16000 Algérie; ^2^ Laboratoire Sciences Nucléaires et Interactions Rayonnement‐Matière, Faculté de Physique USTHB BP 32 El‐Alia, 16123 Bab Ezzouar Algiers Algeria

**Keywords:** absorbed dose to water, calibration coefficient, $N_{D,w}$, water phantom, solid phantom

## Abstract

In the International Atomic Energy Agency's (IAEA) code of practice (TRS 398) and the American Association of Physicists in Medicine's dosimetry protocol (TG‐51), full‐scatter water phantoms are recommended for the determination of the absorbed dose for both photon and electron beams and, consequently, for the calibration of the user's ionization chambers. This procedure is applied in the Secondary Standard Dosimetry Laboratory, where the calibration is performed on a C60o gamma beam, in comparison with reference chambers whose absorbed dose‐to‐water calibration coefficients, $N_{D,w}$, are known. In this work, we present the results of the calibration of 10 Farmer‐like ionization chambers calibrated in three water phantoms (sizes $20\times 20\times 15\;{\text{cm}}^{3}$, $30\times 30\times 30\;{\text{cm}}^{3}$, and $35\times 35\times 37\;{\text{cm}}^{3}$) and two plastic phantoms (size $20\times 20\times 20\;{\text{cm}}^{3}$) polymethyl methacrlyate (PMMA) and polystyrene). Calibrations are performed by the substitution method using an ionization chamber whose $N_{D,w}$ has been supplied by the IAEA's reference laboratory. It is shown that the results, expressed as the percentage ratio of the calibration coefficient in a given phantom to that of the standard IAEA phantom, is less than 0.35% for all investigated chambers, and that the standard deviation of the mean of the $N_{D,w}$ calibration coefficients determined in all five phantoms is less than 0.06%, except for one nylon‐walled ionization chamber, where the observed 0.34% value could be explained by the hygroscopic properties of nylon. Furthermore, a chamber‐to‐chamber dependence of the calibration coefficient has been shown to vary by up to 2.8%. These results emphasize that the phantom dimensions and its material are not sensitive criteria for the calibration of cylindrical ionization chambers in terms of absorbed dose to water. The results also show that generic calibration coefficients could not be considered for a given type of chamber.

PACS number: 87.53.Dq

## I. INTRODUCTION

It has been established that the required accuracy of the absorbed dose delivered to the target volume in radiotherapy is considerably improved if the calibration of a clinical beam is performed with ionization chambers calibrated in terms of absorbed dose to water.^(1)^ In this context, the recent dosimetry protocols published by the International Atomic Energy Agency^(2)^ (IAEA) and the American Association of Physicists in Medicine^(3)^ (AAPM) are based on the use of an ionization chamber calibrated in terms of absorbed dose to water in a standard laboratory's reference quality beam, generally taken as a C60o gamma ray beam.

Both cylindrical and plane‐parallel ionization chambers are recommended as reference instruments for the calibration of the user's C60o gamma ray beam, and water is recommended as the reference medium for absorbed dose measurements.

The accuracy and traceability of the calibration of radiotherapy dosimeters are of great concern, since absorbed dose cannot be obtained with non‐calibrated instruments. The IAEA's code of practice recommends that the calibration coefficient used to determine the absorbed dose be obtained in a water phantom. The recommended water phantoms should be full‐scatter ones extending at least 5 cm beyond all four sides of the largest field size used at the depth of measurement and with at least 5 cm beyond the maximum depth of measurement.

In this work, we investigate the influence of the phantom size and its material over the calibration coefficient. For this purpose, 10 Farmer‐like ionization chambers were calibrated in five different phantoms using the substitution method, and the calibration coefficients were compared to those obtained in the IAEA standard water phantom.

## II. MATERIALS AND METHODS

### A. Experimental equipment and reference conditions

#### A.1 The Secondary Standard Dosimetry Laboratory (SSDL) C60o unit

**Figure 1:**
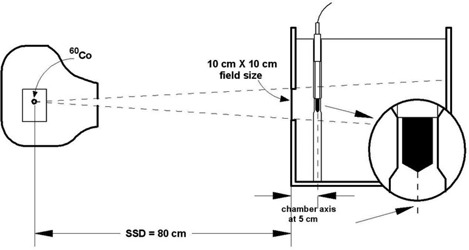
The geometrical conditions used for the calibrations of the ionization chambers in terms of absorbed dose to water. (The is for a water phantom.)

Calibrations were performed in C60o gamma beams using an Eldorado 78 unit. The output in terms of absorbed dose to water was 1120 mGy/min, at a source‐to‐surface distance (SSD) of 80 cm, a depth of 5 cm, and a field size of $10\times 10\;{\text{cm}}^{2}$ at the phantom surface (Fig. 1). The beam flatness at these conditions is about 0.3% within ±4 cm of the beam axis.

#### A.2 Ionization chambers

Five types of commonly used ionization chambers were provided by radiotherapy centers for the present work, giving an overall total of 10 chambers. The characteristics of these chambers are shown in Table 1. Among these chambers, six are the widely used Farmer NE 2571 chamber. The WDIC70 ionization chamber, manufactured by Wellhöfer, is inherently waterproof. However, for measurements in water, all the chambers are used with a 1‐mm PMMA waterproofing sleeve. The reference chamber WDIC70 #141 was calibrated at the IAEA's reference laboratory. The calibration coefficient $\left(N_{D,w}=47.9\pm 0.2\;\text{mGy}/\text{nC}\right)$, established at the conditions T=20 °C and P=101.325 kPa, is thus traceable to the Bureau International des Poids et Mesures (International Bureau of Weights and Measures).

**Table 1:** Characteristics of the cylindrical ionization chambers used in the present study

		Ionization chamber type				
		PTW 30001	PTW 30004	NE 2571	NE 2581	WDIC 70
cavity	volume (cm^3^)	0.6	0.6	0.6	0.6	0.67
	length (mm)	23	23	24	24	23
	radius (mm)	3.1	3.1	3.2	3.2	3.1
wall	material	PMMA	graphite	graphite	A‐150	graphite
	Thickness $\left(g/{\text{cm}}^{2}\right)$	0.045	0.079	0.065	0.041	0.068
central electrode		Al	Al	Al	A‐150	Al
waterproof		N	N	N	N	Y
polarizing voltage (V)		−400	−400	−250	−250	−250
number of chambers		1	1	6	1	1

#### A.3 Current measurements

The reference ionization current was measured with a Keithley 6517 electrometer, and the chambers listed in Table 1 were calibrated with a PTW UNIDOS 10002 electrometer. The time required by the measuring assembly to stabilize was investigated with the Keithley electrometer along with all the chambers. Current measurements were collected at regular intervals after switching on the system, by means of a GPIB computer interface. The results showed that within the first 30 min, the signal varied up to 0.5% for all chambers and could reach 1% for the NE 2581 chamber. Therefore, the measuring assemblies were left at least 2 h before carrying out any measurements. The leakage currents were found to be less than 0.01% of the typical measured currents. The calibration factors of the electrometers were checked with a current source of the Keithley 263 type and were found to be within 0.03% of unity for both electrometers. The recombination and polarity effects were checked and were found to be negligible for all the studied chambers. Thus, the measured currents were not corrected for these effects.

#### A.4 Calibration phantoms

The calibrations were carried out in three water phantoms of different sizes and two plastic phantoms (PMMA and polystyrene). The characteristics of these phantoms are given in Table 2.

**Table 2:** Characteristics of the phantoms used for the calibrations in terms of absorbed dose to water

Model	Material phantom+window	Size L×W×H (cm^3^)	Window thickness (mm)
IAEA standard phantom	water+PMMA	30×30×30	2.5
QC (NE2528/3A)	water+PMMA	20×20×15	2.5
Mylar window phantom	water+Mylar	35×35×37	0.3
PMMA	PMMA	20×20×20	—
polystyrene	polystyrene	20×20×20	—

#### A.5 Geometrical conditions

The calibrations were carried out with the geometrical axis of the chambers situated at a depth of 5 cm. The SSD was 80 cm, and the field size at that distance was $10\times 10\;{\text{cm}}^{2}$ (Fig. 1). All the calibrations were performed using a horizontal beam except for the PMMA phantom, which consists of a homogeneous parallelepipedic block fixed on a supporting plate that can be accurately fixed to the collimator of the C60o Eldorado 78 unit using four rigid metallic stems, and in which the ionization chambers can be irradiated vertically in reproducible conditions.

For the IAEA standard phantom and the Mylar window phantom, the chambers were used with the same 1‐mm thick PMMA waterproofing sleeve. For these two phantoms, special attention was paid to the outward bowing of their windows owing to the water pressure on the inner surface. This effect, which occurs as soon as the phantom is filled and which depends on the size of the phantom and the window, can change the chamber depth and the SSD considerably. Indeed, the water pressure is important with larger phantoms, and the deformation is maximal with larger and thinner windows. The actual deformation was monitored with a 0.01‐mm precision comparator and evaluated to be 0.06 mm and 3 mm for the IAEA and Mylar window phantoms, respectively. The SSD and the chamber depth were corrected accordingly; in addition, to avoid any fluctuations, the water level of the two phantoms was controlled and kept constant during the entire period of the work.

The positioning of the chambers in the plastic phantom is done thanks to a bar, made of the same phantom material, and fitting into existing holes centered at a depth of 5 cm in the phantoms. These bars are machined in order to precisely accommodate the ionization chambers, bringing their geometric center at this depth.

### B. Calibration method

The chambers were calibrated in terms of absorbed dose to water using the substitution method. According to this method, the reference chamber is placed at the reference point in the beam, and a set of readings is taken, leading to the mean reading $M_{\text{ref}}$. It is then replaced by the chamber to be calibrated and a similar set of readings is taken, in the same conditions; the mean reading is then $M_{u}$.

The calibration coefficient $N_{D,w}$ is given by

(1) $$N_{D,w}=\frac{D_{w}}{M_{u}^{\text{corr}}},$$

where $D_{w}$ is the absorbed dose to water, determined by the reference chamber, and $M_{u}^{\text{corr}}$ is the corrected reading of the chamber to be calibrated.

According to the TRS398 IAEA code of practice,^(2)^ the absorbed dose to water, $D_{W}$, is determined according to

(2) $$D_{w}=M_{\text{ref}}^{\text{corr}}N_{D,w}^{\text{ref}},$$

where $N_{D,w}^{\text{ref}}$ is the absorbed dose‐to‐water calibration coefficient of the reference chamber obtained from a standard laboratory, and $M_{\text{ref}}^{\text{corr}}$ is its reading corrected for temperature and pressure according to the following equation:

(3) $$M_{\text{ref}}^{\text{corr}}=M_{\text{ref}}\frac{\left(273.15+T\right)}{293.15}\frac{101.325}{P};$$

*T* and *P* are, respectively, the temperature in degrees Celsius and the pressure in kilopascals. The temperature is measured with a classic thermometer inserted in a waterproofing sleeve identical to that of the cylindrical chamber.

The absorbed dose‐to‐water calibration coefficient of the calibrated ionization chambers is thus given by

(4) $$N_{D,w}=M_{\text{ref}}^{\text{corr}}\frac{N_{D,w}^{\text{ref}}}{M_{u}^{\text{corr}}},$$

where $M_{u}^{\text{corr}}$ is calculated according to the same procedure described above.

## III. RESULTS AND DISCUSSIONS

### A. Results of the calibrations

The results of the calibrations are summarized in Table 3. As can be seen, the relative standard deviations of the mean of the calibration coefficients are less than 0.06% (relative standard deviation 0.14%), except for the NE 2581 ionization chamber, where it is 0.34% (relative standard deviation 0.67%).

**Table 3:** Results of the calibrations in all phantoms. The last column represents the standard deviation of the mean, which is obtained by dividing the standard deviation by the square root of N, where N is the number of individual values of $N_{D,w}$ (in this case, N=4 or N=5).

					$N_{D,w}\;(\text{mGy}/\text{nC})$				
	Chambers				Phantoms				
Code	Type	Serial No.	IAEA	QC	Mylar Win	PMMA	Polyst.	Mean	SD of mean
Ch1	PTW 30001 2114	52.570	52.476	52.520	52.529	52.573	52.534	0.03%
Ch2	PTW 30004	208	52.623	52.616	52.572	52.667	52.678	52.631	0.04%
Ch3	NE 2571	1941	45.536	45.401	45.412	45.378	45.382	45.422	0.06%
Ch4	NE 2571	2347	45.603	45.581	45.621	45.616	—	45.605	0.02%
Ch5	NE 2571	2399	46.127	46.201	46.139	46.141	—	46.152	0.04%
Ch6	NE 2571	2400	45.708	45.687	45.745	45.707	45.828	45.735	0.05%
Ch7	NE 2571	2401	45.335	45.416	45.420	45.422	—	45.398	0.05%
Ch8	NE 2571	2402	44.984	44.924	44.945	44.954	—	44.952	0.03%
Ch9	NE 2581	814	56.994	57.766	57.297	57.794	—	57.463	0.34%
Ch10	WDIC70	039	48.106	48.110	48.171	48.111	—	48.124	0.03%

Furthermore, the results are expressed as the relative deviation of the calibration coefficients obtained in a given phantom to the ones obtained in the IAEA phantom according to the following equation:

(5) $$\Delta (\%)=\frac{\left(N_{D,w}^{\text{PH}}-N_{D,w}^{\text{IAEA}}\right)}{N_{D,w}^{\text{IAEA}}}\times 100,$$

where $N_{D,w}^{\text{IAEA}}$ and $N_{D,w}^{\text{PH}}$ are, respectively, the calibration coefficients obtained in the standard IAEA water phantom and in the other phantoms.

**Figure 2:**
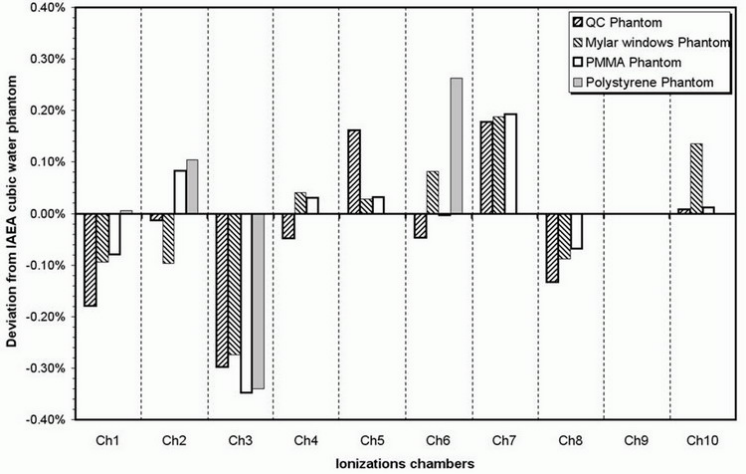
Deviations of the calibration coefficients from the values obtained in the IAEA cubic water phantom.

As can be seen in Fig. 2, these deviations, in absolute values, lie between 0.01% and 0.35%. We have calculated that 35% and 87% of the deviations are less than 0.05% and 0.2%, respectively.

The results are less satisfactory with the NE 2581 ionization chamber, which has not been included in Fig. 2. The wall of this chamber is made of nylon, and, as pointed out by several authors,^(^ ^4^ ^,^ ^5^ ^)^ the response of such chambers is strongly affected by environmental conditions due to the hygroscopic properties of nylon.

### B. Chamber‐to‐chamber variation

**Figure 3:**
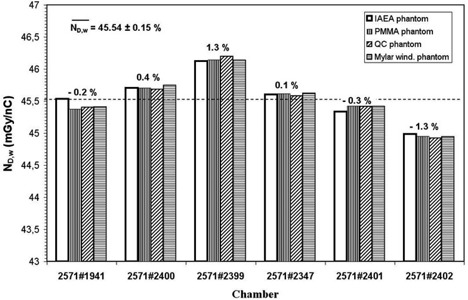
Chamber‐to‐chamber variations of the NE 2571 ionization chamber. The dashed line represents the overall mean calibration coefficient, calculated for all the ionization chambers.

The chamber‐to‐chamber variation of the calibration coefficients is illustrated in Fig. 3 for the NE 2571 chamber. As can be seen, the relative standard deviation of the $N_{D,w}$ mean is 0.15%. This appears to be a good result; however, if we consider the mean values of the calibration coefficients obtained in the four phantoms, the deviation between the maximum and the minimum values is around 2.8%. This stresses the need for individual calibration of each chamber of the same type and allows us to recommend not using generic calibration coefficients. For the polystyrene phantom, only four chambers have been made available; thus the chamber‐to‐chamber study could not be extended for this solid phantom.

### C. Uncertainty analysis

Many factors affect the absorbed dose‐to‐water calibration coefficient. Some of these factors have been identified, and their effect, evaluated in terms of uncertainties, is analyzed. The overall uncertainty is calculated according to the recommendations of the ISO guide for the expression of uncertainty.^(6)^

#### C.1 Type A and type B uncertainties

According to the ISO guide,^(6)^ the uncertainties are classified as type A or type B: A type A uncertainty can be evaluated using statistical techniques. This uncertainty, which measures the repeatability of a result under constant conditions, is assumed to have a normal probability distribution and can be determined by a series of measurements $\left(y_{i}\right)$ in which an estimate, *s*(σ), of the standard deviation, σ, is obtained by a series of *n* measurements, applying

(6) $$s(\sigma )={\left(\frac{\sum {\left(y_{i}-y_{m}\right)}^{2}}{n-1}\right)}^{1/2},$$

where $y_{m}$ is the mean value of *y*.

The standard deviation of the mean, $s(\sigma )/\sqrt{n}$, is the value to be used for the summation in quadrature for obtaining the combined standard uncertainty (see below).

All uncertainties that cannot be determined by a series of repeated measurements are type B uncertainties. They arise from a variety of sources, and the probability distributions may take a variety of shapes. Practical guidance on evaluating type B components is given in Ref. 6.

#### C.2 Combined standard uncertainty

The combined standard uncertainty of the output quantity, *u*(*y*), is derived by the summation in quadrature of all type A and type B standard uncertainties due to the input parameters:

(7) $$u(y)={\left(\sum u_{i}{\left(y\right)}^{2}\right)}^{1/2}.$$

#### C.3 Expanded uncertainty

The overall uncertainty to be quoted for the calibration coefficient is the expanded uncertainty, *U*, which represents the total uncertainty for a specific level of confidence. It is derived by multiplying the combined standard uncertainty, *u*(*y*), by a coverage factor, *k*, which is selected to give the desired level of confidence for a normal distribution. Typical choices for *k* are the integers 1, 2, or 3, which correspond to confidence levels of 67.7%, 95.5%, and 99.7%, respectively. For most radiological applications, a 95% confidence level, for which k=1.96, is recommended. For convenience, this is rounded upwards to a value of exactly 2.0.

#### C.4 Uncertainty over the $N_{D,w}$ calibration coefficient

Table 4 shows the uncertainty budget for the calibration process. Using Eqs. (3) and (4), the following uncertainty contributors can be identified.

**Table 4:** Uncertainty budget for the absorbed dose‐to‐water calibration coefficient

Source of uncertainty	Type A	Type B
1. Factors influencing only the reference standard:		
1.1 Constancy of the dosimeter		0.100
1.2 Dosimeter reading	0.010	0.010
1.3 Temperature *T*: difference with *T* inside the cavity		0.060
Thermometer resolution		0.020
1.4 Pressure		0.060
1.5 Current/charge measurements		0.060
1.6 Reproducibility of the phantom positioning	0.040	
Quadratic sum	0.041	0.144
Combined uncertainty 1	0.150	
2. Factors influencing only the user's chamber		
2.1 Dosimeter reading	0.020	0.060
2.2 Temperature: difference with *T* inside the cavity		0.060
Thermometer resolution		0.020
2.3 Pressure		0.060
2.4 Current/charge measurements		0.060
2.5 Leakage current		0.020
2.6 Reproducibility of the phantom positioning	0.040	
Quadratic sum	0.045	0.108
Combined uncertainty 2	0.117	
3. Total uncertainty		
3.1 Quadratic sum (1+2)	0.061	0.180
3.2 Combined uncertainty SSDL (1+2)	0.190	
3.3 Uncertainty of the calibration coefficient reported by IAEA		0.490
3.4 Combined uncertainty (SSDL+IAEA)	0.526	
3.5 Expanded uncertainty (k=2)	1.052	

### C.4.1 Dosimeter reading

For the reference measurements, a series of 20 readings is taken, and the standard deviation of the mean obtained is of the order 0.01%. The resolution of the reference instruments is equal to 0.02%. Assuming a rectangular distribution, the uncertainty is 0.01%. This uncertainty is taken to be of type B.

### C.4.2 Uncertainty in the calibration coefficient of the standard dosimeter

The uncertainty over the calibration coefficient of the SSDL working standard, taken from the calibration certificate, is reported as a type B uncertainty.

### C.4.3 Constancy of the secondary standard

Constancy of the secondary standard is obtained by evaluating the long‐term stability of the secondary standard system. This uncertainty, estimated from a series of output rate measurements, is dominated by a component having a type A evaluation (standard deviation of the mean of the measurements). Because this component is not directly determined during each measurement, it is treated as a type B evaluation.

### C.4.4 Current/Charge measurements

Typically, the resolution of the therapy level dosimeters is within ±0.1%. This is evaluated as a type B component. Assuming a rectangular distribution, the relative uncertainty is 0.06%.

### C.4.5 Leakage current

The leakage is evaluated before each calibration and is subtracted from the instrument reading. The uncertainty over this factor is evaluated to be 0.02%.

### C.4.6 Effect of the temperature correction factor

For corrections using Eq. (3), the temperature readings are done with a thermometer placed in water or plastic phantom. The difference between the temperature measured by the thermometer and the temperature inside the air cavity is estimated to be less than 0.1%. Assuming a rectangular distribution, the uncertainty (taken as type B) is evaluated to be 0.06%. Another type B component coming from the thermometer resolution (0.1 °C) is included for both measurements (reference and user). Assuming a rectangular distribution, the relative uncertainty is 0.02%.

### C.4.7 Effect of the pressure correction factor

In Eq. (3), the pressure is measured with a classic barometer. When compared with a mercury absolute barometer, its readings are within ±0.1%. Assuming a rectangular distribution, the type B relative uncertainty is 0.06%. The uncertainty component related to the resolution of the barometer is negligible.

### C.4.8 Reproducibility of phantom positioning

This uncertainty, taken as type A, is evaluated to be 0.04%.

As can be seen in Table 4, the overall uncertainty over the calibration coefficient is 1.05%.

## IV. CONCLUSION

Compared to the overall uncertainty over the absorbed dose‐to‐water calibration coefficient, which is around 1.1% (given at 95% confidence level), the difference between the calibration coefficients obtained in this study for the different phantoms and those for all the investigated ionization chambers is not significant. The most important discrepancy is observed for the NE 2581 chamber, whose response is strongly affected by environmental conditions due to the composition of its wall (nylon). Therefore, the deviations observed in this study for this particular chamber was expected.

Our study shows that the type and dimensions of the phantom used are not critical parameters. The absorbed dose‐to‐water calibration coefficients can be determined in any water or solid phantom whose dimensions are at least $20\times 20\times 20\;{\text{cm}}^{3}$. Smaller phantoms were not available during our study; thus, no conclusions could be drawn for these phantoms. The results concerning plastic phantoms do not seem to be consistent with modern dosimetry protocols, which recommend using water phantoms for the calibration of ionization chambers in terms of absorbed dose to water. However, we would like to point out that with the results obtained in this work, we are not advocating the calibration of ionization chambers in plastic phantoms. This should be performed according to the internationally recognized codes of practice.

Furthermore, we have shown an important chamber‐to‐chamber dependence of the calibration coefficients obtained in different phantoms, for the widely used NE 2571 ionization chamber, which demonstrates the individual physical properties of each ionization chamber of the same type and stresses the need of carrying out individual calibrations for each type of ionization chamber.

## AKNOWLEDGMENT

This work was carried out in the framework of an IAEA Coordinated Research project (ALG 11623).
